# Clinical Outcomes of Hematopoietic Cell Transplantation and Chimeric Antigen Receptor T-cell Therapy in Patients With Antecedent *Mycobacterium avium* Complex Pulmonary Disease: A Case Series of 8 Patients

**DOI:** 10.1093/ofid/ofaf268

**Published:** 2025-05-02

**Authors:** Jessica Balbin, Jessica Ferguson Toll, David J Epstein

**Affiliations:** Department of Medicine, Division of Infectious Diseases and Geographic Medicine, Stanford University School of Medicine, Stanford, California, USA; Department of Medicine, Division of Infectious Diseases and Geographic Medicine, Stanford University School of Medicine, Stanford, California, USA; Department of Medicine, Division of Infectious Diseases and Geographic Medicine, Stanford University School of Medicine, Stanford, California, USA

**Keywords:** chimeric antigen receptor (CAR-T) therapy, hematopoietic cell transplantation (HCT), nontuberculous mycobacteria pulmonary disease (NTM-PD), preexisting infection

## Abstract

In this single-center case series, we identified 8 patients with nontuberculous mycobacterial pulmonary disease (NTM-PD), all due to *Mycobacterium avium* complex, who underwent hematopoietic cell transplantation (HCT) or chimeric antigen receptor T-cell (CAR-T) therapy. None, including 3 without NTM-PD treatment before HCT/CAR-T therapy, developed any apparent complications from NTM-PD. This experience suggests that NTM-PD should not preclude HCT/CAR-T therapy.

Hematopoietic cell transplantation (HCT) and chimeric antigen receptor T-cell (CAR-T) therapies are associated with serious infections due to intensive and prolonged immunosuppression. Clinicians are, therefore, appropriately concerned about the safety of these treatments in patients with ongoing or recent infections. While studies have demonstrated deleterious impacts of some pre-HCT/CAR-T infections on subsequent outcomes, other antecedent infections may not substantially affect patient outcomes [1–3]. Understanding nuances in how cellular therapies may affect outcomes with different infections is critical in balancing risks of delaying cancer therapy against risks of uncontrolled infections.

Data on nontuberculous mycobacterial (NTM) pulmonary disease (NTM-PD) before HCT/CAR-T therapy are sparse. One study from over 20 years ago describes 5 patients with apparent NTM-PD before allogeneic HCT, though these patients were excluded from the primary analysis and their cases are described only briefly [4]. Another case of *Mycobacterium kansasii* lung disease before allogeneic HCT was reported from the 1980s [5, 6]. Almost all modern studies on NTM infection in this patient population focus on infections after rather than before these therapies [7, 8]. A minority describe outcomes of allogeneic HCT in patients with inborn errors of immunity affecting the interferon γ–interleukin 12 axis, many of whom had disseminated or other extrapulmonary NTM infections [9, 10]. Many of these studies focus on extrapulmonary NTM disease or combine patients with pulmonary and extrapulmonary NTM disease, although differences in risk factors, pathophysiology, clinical manifestations, imaging, and histopathology are profound enough that NTM-PD and disseminated NTM are, effectively, different diseases entirely [4, 7–12]. To our knowledge, no published data exist on NTM-PD in the setting of CAR-T therapy.

It is unknown whether and how preexisting NTM-PD in other patients affects outcomes after HCT/CAR-T therapy. Given increasing rates of NTM-PD over time, especially among older adults, along with increasingly older patients receiving HCT/CAR-T therapy, this situation will likely be increasingly common [13, 14]. In a single-center cohort of patients receiving HCT/CAR-T therapy, we identify and describe patients with ongoing or recent NTM-PD, diagnosed according to consensus criteria, at the time of HCT/CAR-T therapy.

## METHODS

This study was performed in a large-volume tertiary care hospital in Northern California. Using an institution-wide electronic clinical database, we identified all patients with a possible diagnosis of NTM-PD based on *International Classification of Diseases* codes, administration or prescription of medications primarily used for mycobacterial infections (ethambutol, amikacin, and clofazimine), and laboratory results. We used this same database to identify adult patients who received any commercially available CAR-T product from 2010 through 2023. We identified all adult patients who received allogeneic and autologous HCT at our institution from 2010 to 2023, maintained in a separate comprehensive database. We matched the medical record numbers of patients receiving HCT/CAR-T therapy with those of patients identified as having or likely having NTM-PD.

We reviewed these records and included only patients who had positive cultures or remained on therapy for NTM-PD within a year before HCT/CAR-T therapy. NTM-PD was generally defined by consensus criteria requiring the presence of pulmonary or systemic symptoms, cavitary or nodular/bronchiectatic findings on imaging, and the presence of specific microbiologic criteria. Microbiologic criteria could be fulfilled by growth from ≥2 sputum cultures, growth from 1 bronchial wash or bronchoalveolar lavage (BAL) fluid culture, or compatible histopathology with growth from ≥1 respiratory culture [15]. Although most patients with noncavitary NTM-PD have bronchiectasis, a significant minority do not, so we also included patients with tree-in-bud pattern or centrilobular nodules, even if they did not have bronchiectasis [16, 17]. Since the specificity of lung tissue culture (typically from transbronchial biopsy) is at least as high as that of bronchial wash or BAL fluid specimens, we considered these specimens equivalent.

We excluded patients with cultures identifying *Mycobacterium gordonae*, as this organism is almost never pathogenic [18]. We also excluded *Mycobacteroides chelonae* or *Mycolicibacterium fortuitum* from respiratory tract specimens unless identified from ≥2 cultures obtained via bronchoscopy ≥1 week apart within the same year or unless identified from a biopsy specimen with compatible histopathology. These stringent criteria for *M chelonae* and *M fortuitum* were designed to increase diagnostic specificity, given the infrequency with which these organisms cause NTM-PD and the understanding that NTM-PD diagnostic criteria are not well established for all NTM species [15, 19]. We also excluded patients with inborn errors of immunity, including those undergoing transplantation for neoplasms associated with a *GATA2* mutation. Using electronic medical records and imaging databases, we reviewed the laboratory, imaging, and clinical courses of patients with antecedent NTM-PD. Patients were followed up from the date of a positive culture to the time of study completion (fall 2024). The Stanford University Research Compliance Office approved the study on 27 November 2023 (IRB-73069).

## RESULTS

**Figure 1.**
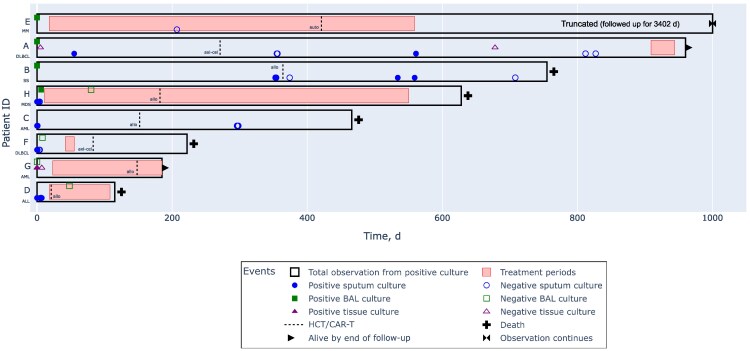
Swimmer plot of diagnosis, treatment, and outcomes. Abbreviations: ALL, acute lymphoblastic leukemia; allo, allogeneic [HCT]; AML, acute myeloid leukemia; auto, autologous [HCT]; axi-cel, axicabtagene ciloleucel; BAL, bronchoalveolar lavage; CAR-T, chimeric antigen receptor T-cell; DLBCL, diffuse large B-cell lymphoma; HCT, hematopoietic cell transplantation; ID, identifier; MDS, myelodysplastic syndrome; MM, multiple myeloma; SS, Sézary syndrome.

Among 5537 patients who received CAR-T therapy or allogeneic or autologous HCT from 2010 to 2023, we identified 8 (0.14%) with NTM-PD who met inclusion criteria (Supplementary Figure 1). They had a variety of hematologic cancers; 5 were treated with allogeneic HCT, 1 with autologous HCT, and 2 with CAR-T therapy (Table 1). The only organism involved was *Mycobacterium avium* complex (MAC) (Table 2). Patient histories are provided below and in Figure 1. The diagnostic criteria patients fulfilled to establish a diagnosis of NTM-PD are described in Table 3.

**Table 1.** Baseline Clinical and Treatment Characteristics

Patient ID	Patient Age, y/Sex	Disease	Cellular Therapy	Conditioning Regimen	GVHD Prophylaxis
A	70/F	DLBCL	CAR-T therapy (axi-cel)	Bendamustine	…
B	74/F	SS	Allogeneic HCT (MUD, PB)	TSEBT/TLI/ATG (NMA)	FK/MMF
C	71/M	AML	Allogeneic HCT (haplo, PB)	Flu/Cy/TBI (RIC)	FK/MMF/Cy
D	36/F	ALL	Allogeneic HCT (UCB)	Flu/Cy/fTBI (MA)	CsA/MMF
E	55/F	MM	Autologous HCT	Mel	…
F	38/M	DLBCL	CAR-T therapy (axi-cel)	Flu/Cy	…
G	62/F	AML	Allogeneic HCT (MUD, PB)	Flu/Mel (RIC)	FK/MMF/Cy
H	68/F	MDS	Allogeneic HCT (MUD)	TLI/ATG (NMA)	CsA/MMF

Abbreviations: ALL, acute lymphoblastic leukemia; AML, acute myeloid leukemia; ATG, anti-thymocyte globulin; axi-cel, axicabtagene ciloleucel; CAR-T, chimeric antigen receptor T-cell; CsA, cyclosporine A; Cy, cyclophosphamide; DLBCL, diffuse large B-cell lymphoma; F, female; FK, tacrolimus; Flu, fludarabine; fTBI, fractionated total-body irradiation; GVHD, graft-vs-host disease, haplo, haploidentical; HCT, hematopoietic cell transplantation; ID, identifier; M, male; MA, myeloablative; MDS, myelodysplastic syndrome; Mel, melphalan; MM, multiple myeloma; MMF, mycophenolate mofetil; MUD, matched unrelated donor; NMA, nonmyeloablative; PB, peripheral blood; RIC, reduced-intensity conditioning; SS, Sézary syndrome; TLI, total lymphoid irradiation; TSEBT, total skin electron beam therapy; UCB, umbilical cord blood.

**Table 2.** Underlying Lung Disease and Characteristics of Nontuberculous Mycobacterial Pulmonary Disease Diagnosis, Imaging, and Treatment

Patient ID	Lung Disease	Organism	Smear Positive	Cavitary Disease	Treatment
A	None	MAC	Yes	No	None
B	COPD; bronchiectasis	MAC	No	No	None
C	None	MAC	No	No	None
D	None	MAC	No	Yes	Azithromycin + EMB ± FQ
E	None	MAC	No	Yes	Azithromycin + EMB ± RIF
F	None	MAC	No	Yes	Azithromycin + EMB + rifabutin
G	None	MAC	No	No	Azithromycin + EMB ± rifabutin
H	Bronchiectasis	MAC	Yes	No	Azithromycin + EMB ± RIF

Abbreviations: COPD, chronic obstructive pulmonary disease; EMB, ethambutol; FQ, fluoroquinolone; HCT, hematopoietic cell transplantation; ID, identifier; MAC, *Mycobacterium avium* complex; RIF, rifampin.

**Table 3.** Diagnostic Criteria for Nontuberculous Mycobacterial Pulmonary Disease

Patient ID	Microbiologic Criteria	Radiographic Criteria	Clinical Criteria
A	Histopathology with granulomatous inflammation; MAC from BAL fluid and sputum cultures	Tree-in-bud pattern; consolidation	Chronic cough
B	MAC from BAL fluid and sputum cultures	Tree-in-bud pattern; bronchiectasis	Chronic cough; dyspnea
C	MAC from 2 sputum cultures	Tree-in-bud pattern	Cough; hemoptysis; fever
D	MAC from 2 sputum cultures	Cavitation	Cough; fever; weakness
E	MAC from BAL culture	Cavitation	Chronic cough
F	MAC from 3 sputum cultures	Cavitation	Cough; dyspnea
G	Histopathology with AFOP; MAC from lung tissue	Consolidation; nodules	Cough; fever
H	MAC from BAL fluid and sputum cultures	Tree-in-bud pattern; bronchiectasis	Cough; fatigue

Abbreviations: AFOP, acute fibrinous and organizing pneumonia; BAL, bronchoalveolar lavage; ID, identifier; MAC, *Mycobacterium avium* complex.

Patient A, a 70-year-old woman with diffuse large B-cell lymphoma (DLBCL) involving lung and bone marrow, had a diagnosis of MAC pulmonary disease (MAC-PD) based on tree-in-bud pattern on chest computed tomography (CT), 1 positive culture from BAL fluid and later from sputum, and the presence of chronic cough. CT-guided transthoracic needle biopsy of an area of consolidation demonstrated granulomatous inflammation on histopathology; scattered small nodules were also seen. Given stable imaging findings and minimal symptoms, the patient was followed up without MAC-PD treatment for approximately 9 months, during which time she was treated with cyclophosphamide, doxorubicin, vincristine, prednisone, and rituximab, until infusion of axicabtagene ciloleucel (axi-cel). Since CAR-T infusion, she has been followed up for approximately 2 years with intermittently positive sputum cultures (including a smear-positive sputum culture). She briefly underwent treatment toward the end of the study period, although treatment was discontinued given dramatic short-interval radiographic improvement which suggested against the presence of significant MAC-PD.

Patient B, a 74-year-old woman with Sézary syndrome, chronic obstructive pulmonary disease treated with long-term oxygen therapy, and bronchiectasis, had a diagnosis of smear-negative nodular bronchiectatic MAC-PD based on a positive culture from BAL fluid, and later, from sputum, in the setting of dyspnea and chronic cough. Over the following year, she was monitored without MAC-PD treatment, given her radiographic stability and absence of symptoms, while she received treatment for Sézary syndrome before undergoing HCT with nonmyeloablative conditioning while still culture positive. She remained intermittently culture positive for a year after HCT, with continued radiographic stability, until she died of complications from gangrenous cholecystitis, chronic obstructive pulmonary disease, and persistent/recurrent lymphoma; autopsy showed no evidence of infection.

Patient C, a 71-year-old man with acute myeloid leukemia (AML), had a diagnosis of MAC-PD based on right upper lobe tree-in-bud pattern and 2 sputum specimens growing MAC, with cough, hemoptysis, and fever. Since the imaging findings were stable and the patient was asymptomatic by the time cultures were available, he was not treated for MAC-PD, and, approximately 5 months later, underwent HCT from a haploidentical donor with reduced-intensity conditioning. He ultimately died of persistent/recurrent AML about 10 months after HCT, by which point spontaneous culture conversion had occurred.

Patient D, a 36-year-old woman with acute lymphoblastic leukemia, had a diagnosis of smear-negative cavitary MAC-PD based on 2 positive sputum cultures, abnormal imaging findings, and the presence of cough, fever, and weakness. She began treatment with azithromycin and ethambutol (along with ciprofloxacin and moxifloxacin for part of the treatment course) and, a few days later, underwent allogeneic HCT using umbilical cord blood with myeloablative conditioning. She was also treated with voriconazole and posaconazole; the small cavity improved on follow-up imaging. She died about 3 months after HCT due to acute respiratory distress syndrome of unclear etiology with diffuse consolidation on chest CT. She was treated empirically for *Pneumocystis jirovecii* pneumonia because elevated *β*-d-glucan; rhinovirus was also detected from polymerase chain reaction of a nasopharyngeal specimen. She was unable to safely undergo BAL at that time, and no autopsy was performed, though a prior BAL fluid culture showed culture conversion early in treatment.

Patient E, a 55-year-old woman with multiple myeloma, had a diagnosis of cavitary smear-negative MAC-PD based on a positive BAL fluid culture and chronic cough. She was started on treatment with azithromycin, ethambutol, and, for most of the treatment course, rifampin. About 1 year later, she underwent autologous HCT, after which treatment continued for several more months. There was limited microbiologic follow-up, but the patient had radiographic and clinical improvement without concerning developments >5 years later.

Patient F, a 38-year-old man with DLBCL, had a diagnosis of cavitary smear-negative MAC-PD with 3 positive sputum cultures and cough and dyspnea. He started treatment with azithromycin, ethambutol, and rifabutin, but all medications were discontinued within 1 month when subsequent negative BAL fluid cultures returned. Moreover, there were potential alternative explanations for the patient’s cavitary lung disease, namely *Staphylococcus aureus* bacteremia with possible septic emboli, though *S aureus* was never recovered from respiratory specimens. He was also treated empirically with voriconazole, though there was no microbiologic evidence of mold infection. Shortly thereafter, the patient received axi-cel. He died of persistent/recurrent DLBCL approximately 5 months after CAR-T therapy, without clinical or radiographic evidence of MAC-PD.

Patient G, a 62-year-old woman with AML, had a diagnosis of MAC-PD based on a lung nodule biopsy with positive culture and accompanying cough and fever. She started azithromycin, ethambutol, and rifabutin initially, though rifabutin was discontinued after 3 months; she was also treated with liposomal amphotericin B, and later isavuconazonium sulfate. She underwent allogeneic HCT with reduced-intensity conditioning after approximately 5 months of antibiotics. She continued antibiotics for 5 months before the end of observation, with radiographic improvement.

Patient H, a 68-year-old woman with bronchiectasis and myelodysplastic syndrome, had a diagnosis of smear-positive noncavitary MAC-PD with 1 positive BAL fluid and 2 positive sputum cultures, along with cough and fatigue. She started treatment with azithromycin, ethambutol, and rifampin shortly thereafter, though rifampin was discontinued within 2 months, at which time repeated BAL resulted in a negative BAL fluid culture. She was also treated with voriconazole and liposomal amphotericin B. Imaging findings improved on serial CT scans. Approximately 6 months after starting antibiotics, the patient underwent allogeneic HCT with nonmyeloablative conditioning. She died >1 year after HCT due to AML.

## DISCUSSION

This case series describes characteristics and outcomes of 8 patients with NTM-PD according to consensus diagnostic criteria antecedent to HCT/CAR-T therapy. MAC was the only organism identified in these patients. Five patients received an azithromycin- and ethambutol-based regimen for varying durations before HCT/CAR-T therapy, ranging from a few days to approximately 1 year. Despite concerns that intensive immunosuppression can exacerbate preexisting infections, we saw no cases of serious complications, such as significant progression of NTM-PD, regardless of treatment status. As with the larger population of patients with NTM-PD, spontaneous culture conversion occurred in some patients [20–22]. The deaths in this group were due to causes unfortunately common in this setting, mostly persistent or relapsed disease, and none related to NTM-PD.

While these data cannot confidently exclude an increased risk of progressive NTM-PD after HCT/CAR-T therapy, they still provide useful information and some reassurance given scant literature on this topic. European guidelines, citing prior published studies, describe high mortality rates in a closely related population of patients with NTM-PD after, rather than before, HCT [23]. However, many of the studies cited to support this claim are problematic for one or more of these reasons: combining cases of pulmonary and extrapulmonary NTM, sometimes in ways that prevent disentangling data for these different groups; classifying as NTM-PD patients with mycobacteremia who clearly have disseminated disease; including as causes of lung disease *M chelonae*, *M fortuitum*, *M gordonae*, and other rare causes of NTM-PD without sufficient information to justify their inclusion; lacking species-level identification of organisms; and providing limited follow-up data [4, 6, 24–27].

Two cited studies do provide useful insights into NTM-PD after HCT, however. One study of South Korean HCT recipients describes 25 cases in which NTM were cultured from respiratory specimens; among the 10 patients treated for NTM-PD, the deaths of 2 were “‘NTM related,’’ compared with 1 such death in untreated patients [28]. Another informative study from Canada described 18 patients with NTM-PD, excluding 1 patient each with disseminated disease and *M gordonae* from BAL fluid culture, 8 of whom died within a year after NTM-PD, though no deaths were apparently due to NTM-PD [29]. Most patients in these studies had graft-vs-host disease, and at least half had graft-vs-host disease of the lung, itself associated with high mortality rates [30, 31] In summary, there is no compelling evidence that NTM-PD significantly affects the mortality risk in this patient population. In our cohort including patients before receipt of HCT/CAR-T therapy, we found a similarly high mortality rate, but again from causes other than NTM-PD.

The pathophysiology of NTM-PD may explain why even profound immune impairment due to both underlying hematologic disease and HCT/CAR-T therapy may not dramatically affect the natural history of NTM-PD. Specifically, key host defenses against NTM-PD are local to the lung, namely structurally normal airways and normal mucociliary function [32]. In fact, while structural lung disease is a potent risk factor for NTM-PD, systemic immunosuppression (excluding tumor necrosis factor α inhibitors) has not been convincingly associated with an increased risk of NTM-PD [33].

Our results and conclusions apply only to NTM-PD. Extrapulmonary NTM, including disseminated NTM, typically due to slow-growing mycobacteria, and catheter-related bloodstream infections, often caused by rapidly growing mycobacteria are separate diseases in all relevant aspects. For example, disseminated infections due to slow-growing NTM are associated with a poor prognosis and require prolonged therapy [34]. And unlike in NTM-PD, cellular immunity is critical in preventing and controlling disseminated NTM infections, so there is reason to be concerned about the impact of conditioning regimens before HCT and lymphodepletion chemotherapy before CAR-T therapy. Despite the concern sometimes raised that untreated NTM-PD can lead to disseminated disease, we have not seen this in our clinical practice focusing on treating NTM in immunocompromised patients, and, as previously mentioned, pulmonary and disseminated NTM infection are fundamentally pathophysiologically different. For example, when disseminated NTM involves the lung, imaging often shows a miliary pattern or consolidations and histopathology shows poorly formed granulomas or mycobacterial pneumonia, in contrast to the bronchocentric imaging findings of NTM-PD with robust necrotizing granulomatous inflammation [35].

The current study is strengthened by robust follow-up of patients, availability of detailed microbiologic and other details, strict inclusion criteria, and a relatively homogeneous patient population including only those with NTM-PD (excluding other forms of NTM disease) over a relatively narrow time period. Moreover, this is the first description to our knowledge of outcomes of patients with NTM-PD undergoing CAR-T therapy. Limitations of the study include challenges associated with diagnosis of NTM-PD in this patient population and issues of confounding inherent to retrospective observational studies. While these patients met consensus criteria for MAC-PD, this does not mean that they truly had MAC-PD [19, 36]. The specificity and sensitivity of these diagnostic criteria are limited by, among other factors, diverse radiographic presentations, difficulty in attributing the causes of patients’ pulmonary and systemic symptoms, uneven performance of microbiologic criteria across species, and the intensity and frequency with which clinicians pursue microbiologic testing [16, 17]. The accuracy of these criteria in patients before HCT/CAR-T therapy is unknown, and there is reason to be thoughtful in their application.

Systemic symptoms like fatigue and weight loss are expected in this population. Some radiographic findings, particularly cavitation, may be more likely due to infection from mold than NTM [37]. The tree-in-bud pattern, on the other hand, indicates airway spread of infection characteristic of NTM but rarely associated with mold infections in this patient population, though tree-in-bud pattern can be seen with airway spread of mold after lung transplant [37]. These challenges in establishing a diagnosis of NTM-PD in the setting of hematologic cancers is further discussed in consensus guidelines [23]. Notably, studies have found broadly similar clinical outcomes among patients with isolation of NTM, regardless of whether or not they formally met consensus criteria for NTM-PD [38]. The retrospective observational nature of this study also introduces challenges in inferring relationships between patient factors and clinical outcomes. For example, as expected, patients with cavitary disease were all treated. Other differences in patient management—specifically whether patients were treated or not—may have reflected provider practices rather than patient characteristics. Moreover, despite the narrow inclusion criteria, patients were still heterogenous in terms of treatment decisions and time from NTM-PD diagnosis to HCT/CAR-T therapy.

There are scant data available on outcomes of HCT with preexisting NTM-PD, and none describing outcomes of CAR-T therapy after NTM-PD. In this single-center case series of 8 patients, including 3 untreated patients, we found no cases of significant progression or clinical consequences of NTM-PD, regardless of treatment status. While these data are limited and cannot rule out the possibility that HCT/CAR-T therapy can lead to progression of NTM-PD, they are nonetheless reassuring. As NTM-PD and hematologic cancers are both increasing in incidence over time, we expect to see more of these comorbid conditions.

## Supplementary Material

ofaf268_Supplementary_Data

## Supplementary Data

Supplementary materials are available at *Open Forum Infectious Diseases* online. Consisting of data provided by the authors to benefit the reader, the posted materials are not copyedited and are the sole responsibility of the authors, so questions or comments should be addressed to the corresponding author.

## Notes

***Acknowledgments*.** We thank Amelia Scheck of the Division of Blood and Marrow Transplantation and Cellular Therapy for assistance with querying and obtaining data from the division's databases.

***Author contributions.*** Study design: J. B. and D. J. E. Data collection: J. B. and J. F. T. Analysis: J. F. T. and D. J. E. Manuscript preparation: All authors.

***Potential conflicts of interest.*** All authors: No reported conflicts.
