# Supplementary material for: Clinical Outcomes of Hematopoietic Cell Transplantation and Chimeric Antigen Receptor T-cell Therapy in Patients With Antecedent Mycobacterium avium Complex Pulmonary Disease: A Case Series of 8 Patients
Source: Open Forum Infect Dis. 2025 May 2;12(5):ofaf268. doi: 10.1093/ofid/ofaf268 (PMC12100489; doi:10.1093/ofid/ofaf268)
Supplement: ofaf268_Supplementary_Data [file ofaf268_supplementary_data.docx]

## Supplement

Allogeneic HCT episodes N = 2,546

Autologous HCT episodes N = 2,564

Patients with allogeneic HCT Patients with autologous HCT N = 2,446 N = 2,564

Total patients N = 5,777

Unique patients N = 5,537

CAR-T episodes N = 667

Final included patients N = 8

Excluded:

No NTM (N = 5,468)

Post-HCT/CAR-T NTM (N = 51)

Extrapulmonary disease (N = 5)

Not meeting diagnostic criteria (N = 2) *Mycobacterium gordonae* (N = 1) Remote NTM (N = 1)

Other (N = 1)

Excluded:

Duplicates (N = 240)

Patients with CAR-T N = 667

Excluded:

Duplicates (N = 100)

Figure S1: STROBE flow diagram.

**Abbreviations:** CAR-T = chimeric antigen receptor T-cell therapy; HCT = hematopoietic cell transplant; NTM = nontuberculous mycobacteria
